# Role of [^18^F]FDG PET/CT in patients with invasive breast carcinoma of no special type: Literature review and comparison between guidelines

**DOI:** 10.1016/j.breast.2024.103806

**Published:** 2024-09-12

**Authors:** David Groheux, Sofia C. Vaz, Philip Poortmans, Ritse M. Mann, Gary A. Ulaner, Gary J.R. Cook, Elif Hindié, John Patrick Pilkington Woll, Heather Jacene, Isabel T. Rubio, Marie-Jeanne Vrancken Peeters, Elizabeth H. Dibble, Lioe-Fee de Geus-Oei, Stephanie L. Graff, Fatima Cardoso

**Affiliations:** a Department of Nuclear Medicine, Saint-Louis Hospital, Paris, France; b University Paris-Diderot, INSERM, U976, Paris, France; c Centre d’Imagerie Radio-Isotopique (CIRI), La Rochelle, France; d Department of Nuclear Medicine and Radiopharmacology, Champalimaud Clinical Center, Champalimaud Foundation, Lisbon, Portugal; e Department of Radiology, Leiden University Medical Center, Leiden, the Netherlands; f Department of Radiation Oncology, Iridium Netwerk, Belgium; g Faculty of Medicine and Health Sciences, University of Antwerp, Wilrijk-Antwerp, Belgium; h Department of Radiology, Radboud umc, Nijmegen, the Netherlands; i Department of Molecular Imaging and Therapy, Hoag Family Cancer Institute, Newport Beach, CA, United States; j Departments of Radiology and Translational Genomics, University of Southern California, Los Angeles, CA, United States; k Department of Cancer Imaging, King's College London, London, UK; l King's College London and Guy's & St Thomas' PET Centre, London, UK; m School of Biomedical Engineering and Imaging Sciences, King's College London, London, UK; n Department of Nuclear Medicine, Bordeaux University Hospital, Bordeaux, France; o Department of PET-CT, Clinica Delgado AUNA, Lima, Peru; p Dana-Farber Cancer Institute/Brigham and Women's Hospital, and Harvard Medical School, United States; q Department of Breast Surgical Oncology, Clinica Universidad de Navarra, Madrid, Cancer Center Clinica Universidad de Navarra, Spain; r Department of Surgical Oncology, Netherlands Cancer Institute, Amsterdam, the Netherlands; s Department of Surgery, Amsterdam University Medical Center, Amsterdam, the Netherlands; t Department of Diagnostic Imaging, The Warren Alpert Medical School of Brown University, Providence, RI, United States; u Biomedical Photonic Imaging Group, University of Twente, Enschede, the Netherlands; v Department of Radiation Science & Technology, Delft University of Technology, Delft, the Netherlands; w Lifespan Cancer Institute, Providence, RI, United States; x Legorreta Cancer Center at Brown University, Providence, RI, United States; y Breast Unit, Champalimaud Clinical Center, Champalimaud Foundation, Lisbon, Portugal

**Keywords:** Breast cancer, FDG-PET/CT, EANM-SNMMI guidelines, NCCN guidelines, ESMO guidelines, ABC guidelines

## Abstract

**Purpose:**

The recently released EANM/SNMMI guideline, endorsed by several important clinical and imaging societies in the field of breast cancer (BC) care (ACR, ESSO, ESTRO, EUSOBI/ESR, EUSOMA), emphasized the role of [^18^F]FDG PET/CT in management of patients with no special type (NST) BC. This review identifies and summarizes similarities, discrepancies and novelties of the EANM/SNMMI guideline compared to NCCN, ESMO and ABC recommendations.

**Methods:**

The EANM/SNMMI guideline was based on a systematic literature search and the AGREE tool. The level of evidence was determined according to NICE criteria, and 85 % agreement or higher was reached regarding each statement. Comparisons with NCCN, ESMO and ABC guidelines were examined for specific clinical scenarios in patients with early stage through advanced and metastatic BC.

**Results:**

Regarding initial staging of patients with NST BC, [^18^F]FDG PET/CT is the preferred modality in the EANM-SNMMI guideline, showing superiority as a single modality to a combination of contrast-enhanced CT of thorax-abdomen-pelvis plus bone scan in head-to-head comparisons and a randomized study. Its use is recommended in patients with clinical stage IIB or higher and may be useful in certain stage IIA cases of NST BC. In NCCN, ESMO, and ABC guidelines, [^18^F]FDG PET/CT is instead recommended as complementary to conventional imaging to solve inconclusive findings, although ESMO and ABC also suggest [^18^F]FDG PET/CT can replace conventional imaging for staging patients with high-risk and metastatic NST BC. During follow up, NCCN and ESMO only recommend diagnostic imaging if there is suspicion of recurrence. Similarly, EANM-SNMMI states that [^18^F]FDG PET/CT is useful to detect the site and extent of recurrence only when there is clinical or laboratory suspicion of recurrence, or when conventional imaging methods are equivocal. The EANM-SNMMI guideline is the first to emphasize a role of [^18^F]FDG PET/CT for assessing early metabolic response to primary systemic therapy, particularly for HER2+ BC and TNBC. In the metastatic setting, EANM-SNMMI state that [^18^F]FDG PET/CT may help evaluate bone metastases and determine early response to treatment, in agreement with guidelines from ESMO.

**Conclusions:**

The recently released EANM/SNMMI guideline reinforces the role of [^18^F]FDG PET/CT in the management of patients with NST BC supported by extensive evidence of its utility in several clinical scenarios.

## Highlights

• The EANM/SNMMI guideline clarifies the role of [18F]FDG PET/CT in patients with breast cancer.
• At initial staging, [^18^F]FDG PET/CT is recommended by the EANM-SNMMI from stage IIB to stage IV.
• In NCCN, ESMO and ABC guidelines, [18F]PET/CT is most often recommended as complementary to conventional imaging. However similarities between EANM-SNMMI, ESMO and ABC are found in the notion that [18F]FDG PET/CT can replace conventional imaging for staging high-risk or metastatic patients with breast cancer, and for early assessment of bone metastases.
• According to EANM-SNMMI, [18F]FDG PET/CT is useful to detect the site and extent of recurrence when there is clinical or laboratory suspicion.
• According to EANM-SNMMI, [18F]FDG PET/CT may play a role in monitoring treatment response, mainly to evaluate bone metastases.

## 1 Introduction

Breast cancer (BC) is the most common cancer among women worldwide, and its diagnosis has been increasing in recent decades [1]. Prognostic information can be obtained from the subtype classification (estrogen receptor (ER), progesterone receptor (PR), human epidermal growth factor receptor 2 receptor [HER2]), tumor stage and, in some cases genomic tests [2]. 2-[^18^F]fluoro-2-deoxy-D-glucose positron emission tomography/computed tomography ([18F]FDG PET/CT) plays an important role in BC staging, and indications in BC management are increasingly recognized. Recently, we collaborated as representatives from the European Association of Nuclear Medicine (EANM) and the Society of Nuclear Medicine and Molecular Imaging (SNMMI) to produce joint European-American guidelines on the role of [^18^F]FDG PET/CT in no special type (NST) BC, endorsed by several other oncology and imaging societies: the American College of Radiology (ACR), the European Society of Surgical Oncology (ESSO), the European Society for Radiotherapy and Oncology (ESTRO), the European Society of Breast Imaging (EUSOBI), the European Society of Radiology (ESR), and the European Society of Breast Cancer Specialists (EUSOMA) [3]. Considering data suggesting lower [^18^F]FDG-avidity and reduced lesion detection in invasive lobular carcinoma (ILC) [[4], [5], [6], [7]], the EANM-SNMMI guidelines were mainly applicable to NST BC. We hereafter review the current roles of [^18^F]FDG PET/CT in BC, including more recent studies, and the points of difference and agreement between the guidelines of the EANM-SNMMI, the American National Comprehensive Cancer Network (NCCN), the European Society for Medical Oncology (ESMO), and the Advanced Breast Cancer International Consensus guidelines (ABC Guidelines). NCCN guidelines are updated topic-wise several times each year, and we refer to "*Clinical Practice Guidelines in Oncology- Breast Cancer- Version 4.2024*" [8]. The ESMO guidelines “*Early breast cancer: ESMO Clinical Practice Guideline for diagnosis, treatment and follow-up,*” first published in 2019 [9], were updated in 2024 [10]. The “*Clinical Practice Guideline for the diagnosis, staging and treatment of patients with metastatic breast cancer*” were published in 2021 [11] and are regularly updated on the ESMO website (ESMO metastatic breast cancer living guidelines [12]). The ABC Consensus Conference develops international consensus guidelines for the management of patients with advanced breast cancer (ABC). The ABC5 guidelines were published in 2020 [13] and were reviewed during the ABC6 meeting and more recently during the ABC7 meeting in November 2023, concluding with a Delphi session and consensus vote, and were published in 2024 [14]. It is important to emphasize that the EANM-SNMMI guideline provides dedicated information about NST BC, while the other clinical guidelines usually refer to PET in breast cancer in general and include some specifications about the best imaging modalities to address the lobular subtype.

## 2 Recommendations from the EANM-SNMMI, NCCN, ESMO and ABC guidelines

### 2.1 Initial workup of breast cancer

**Fig. 1:**
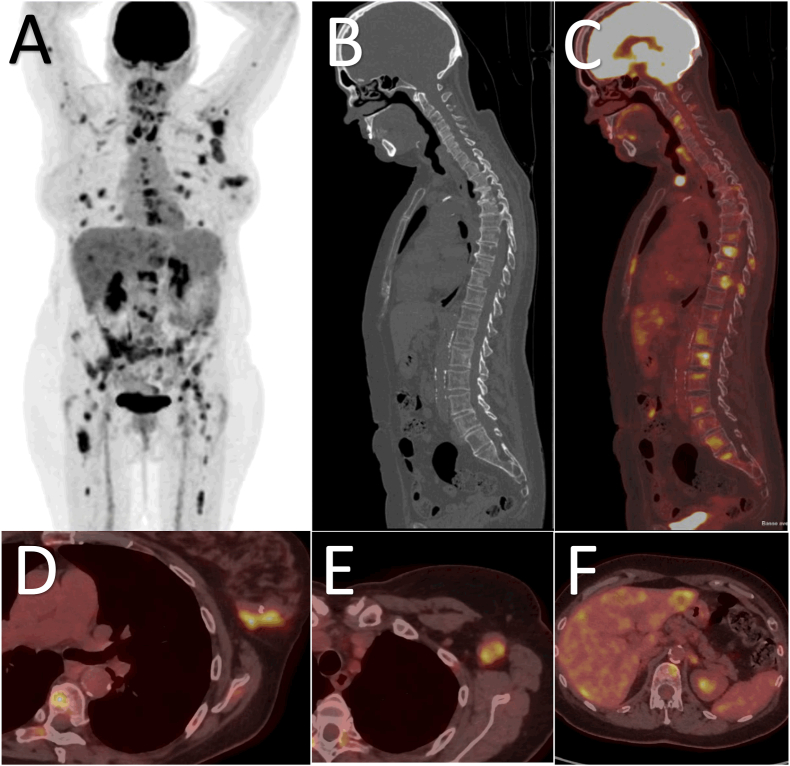
A 67-year-old woman with left NST BC, ER+, PR+, HER2- (luminal A), with clinical axillary lymph nodes was referred for primary staging with [^18^F]FDG PET/CT. MIP PET images (A) showed numerous foci of [^18^F]FDG abnormal uptake. Sagittal view (B and C, CT and PET/CT fusion images) showed numerous bone osteolytic hypermetabolic metastases. Axial PET/CT fusion images showed the primary left breast cancer (D), axillary lymph nodes (E) and liver metastases (F). The disease was classified stage IV.

Pretherapeutic BC staging increasingly incorporates [^18^F]FDG PET/CT [[15], [16], [17]] due to its high accuracy in detecting extra-axillary lymph nodes (LN) and distant metastases, especially in case of locally advanced breast cancer (LABC) or inflammatory BC (T4d) (Table 1) [[18], [19], [20], [21], [22], [23], [24]]. Recently, several studies have shown [^18^F]FDG PET/CT may be useful, not only in patients with LABC, but also in intermediate risk patients [[25], [26], [27], [28], [29], [30], [31], [32], [33], [34], [35], [36]] (Fig. 1).

**Table 1:** Anatomic TNM Stage grouping for Breast Cancer according to the AJCC Cancer Staging Manual [37,38].

AJCC	TNM			Clinical group
**Stage I**	T1^a^	N0	M0	Primary operable breast cancer
**Stage IIA**	T0	N1	M0	
	T1	N1	M0	
	T2	N0	M0	
**Stage IIB**	T2	N1	M0	
	T3	N0	M0	
**Stage IIIA**	T3	N1	M0	
	T0	N2	M0	Locally advanced breast cancer
	T1	N2	M0	
	T2	N2	M0	
	T3	N2	M0	
**Stage IIIB**	T4^b^	N0	M0	
	T4	N1	M0	
	T4	N2	M0	
**Stage IIIC**	any T	N3	M0	
**Stage IV**	any T	any N	M1	Metastatic disease

a T1 is further divided into 4 groups : - T1mi means the cancer is 0.1 cm across or less. - T1a means the cancer is more than 0.1 cm but not more than 0.5 cm. - T1b means the cancer is more than 0.5 cm but not more than 1 cm. - T1c means the cancer is more than 1 cm but not more than 2 cm.

b T4 is divided into 4 groups : -T4a means the cancer has spread into the chest wall. -T4b means the cancer has spread into the skin and the breast might be swollen. -T4c means the cancer has spread to both the skin and the chest wall. -T4d means inflammatory carcinoma.

#### 2.1.1 Systemic staging in early-stage breast cancer, including high- and intermediate-risk patients

In addition to locoregional staging (by mammography, ultrasound ± breast MRI), the NCCN guidelines for BC recommend performing additional workup as follows: T ≥ T2 or N+ disease (i.e. stage IIA and beyond, Table 1), regardless of the BC prognostic subtype. They also suggest additional workup for T1c N0 (i.e., stage I with a primary tumor >1 cm) in case of HER2+ BC or triple negative (ER-/PR-/HER2-) breast cancer (TNBC) [8]. For imaging workup, NCCN recommends a combination of chest CT ± contrast, abdominal ± pelvic CT with contrast (or MRI with contrast) and bone scan or [^18^F]sodium fluoride ([^18^F]NaF) PET/CT (Table 2). In the NCCN guidelines, [^18^F]FDG PET/CT can also be used with this following footnote [8]: “[18F]FDG PET/CT is most beneficial and accurate for advanced disease (stage III) and invasive ductal (compared to ILC) histology, but may be useful in selected circumstances of earlier stage disease (stage IIA disease: T1N1, T2N0) such as: equivocal CT and/or bone scan results; suspicion of undetected nodal and/or distant disease; and treatment response assessment. An [^18^F]FDG PET/CT may be utilized as an adjunct to, or in lieu of, initial standard staging and may be performed simultaneously with diagnostic CT. Conversely, a bone scan or [^18^F]sodium fluoride PET/CT may not be needed if an upfront [18F]FDG PET/CT clearly indicates consistent findings on both PET and CT components.” Joint EANM-SNMMI guidelines also consider [^18^F]FDG PET/CT indicated for stage IIB (T2N1 and T3N0) and higher BC (Table 2), and EANM-SNMMI guidelines recommend [^18^F]FDG PET/CT (instead of, and not in combination with, conventional imaging modalities). According to EANM-SNMMI, [^18^F]FDG PET/CT is also recommended in baseline treatment planning and may improve radiotherapy (RT) planning [3]. These recommendations are based on studies showing [^18^F]FDG PET/CT changes stage in 21 % of patients with stage IIB BC [3]. A good clinical practice guideline (2020) and a meta-analysis (2021) also concluded that [^18^F]FDG PET/CT can be recommended for initial staging to identify distant metastases in patients with clinical stage ≥ IIB BC [16,17]. For stage IIA, EANM-SNMMI guidelines recommend that [^18^F]FDG PET/CT be reserved for specific cases. EANM-SNMMI guidelines do not recommend [18F]FDG PET/CT in workup for stage I BC, regardless of subtype. EANM-SNMMI guidelines do not restrict workup to HER2+ BC or TNBC. Although [^18^F]FDG PET/CT has some limitations for low proliferation, low-grade, and/or well-differentiated luminal tumors, [^18^F]FDG PET/CT imaging is useful for initial BC staging, regardless of tumor phenotype (ER+/HER2-, triple negative, or HER2+) and tumor grade. In a prospective study of 254 patients [30], the rates of extra-axillary LN metastases on [^18^F]FDG PET/CT were higher in grade 3 than low grade tumors (p = 0.004) and in triple negative or HER2+ tumors compared to ER+/HER2- tumors (p = 0.01). However, the rate of distant metastases was not related to tumor grade or BC subtype, which has also been found in other studies [32,39]. The location of metastases differed according to primary tumor subtype: extra-skeletal metastases were more prevalent in HER2+ BC and TNBC [30]. The EANM-SNMMI guidelines consider [^18^F]FDG PET/CT can be used instead of standard initial staging of distant disease (Table 2). In contrast to the NCCN, the EANM-SNMMI guidelines do not require that [^18^F]FDG PET/CT show consistent findings on both PET and CT components to avoid a bone scan or [^18^F]sodium fluoride PET/CT. Morphological changes occur after metabolic changes, and a hypermetabolic focus with normal bone on CT images is a highly suspicious sign of an early bone metastasis [40,41]. Therefore, the EANM-SNMMI expert group does not recommend waiting for changes on CT and does not recommend performing bone scan or 2-[^18^F]NaF PET in addition to [^18^F]FDG PET/CT in this scenario [3].

**Table 2:** Summary of the recommendations for staging patients during the initial imaging workup of breast cancer according to the initial clinical staging.

		Joint EANM-SNMMI Guidelines [3]	NCCN Guidelines [8]	ESMO Guidelines [[9], [10], [11], [12]]	ABC d 5, 6 and 7 Consensus Guidelines [[13], [14], [42]]
Imaging modalities recommended in the systemic staging of non-metastatic BC		[^18^F]FDG PET/CT	• Chest diagnostic CT ± contrast •Abdominal ± pelvic diagnostic CT with contrast or MRI with contrast •Bone scan or sodium fluoride PET/CT •[^18^F]FDG PET/CT^a^	•CT of the chest, abdominal imaging (US, CT or MRI scan) and bone scan can be considered. •[^18^F]FDG PET/CT may be useful when conventional methods are inconclusive. •[^18^F]FDG PET/CT can also replace traditional imaging for staging in high-risk patients.	Not applicable
**Indications according to the stage**	**I (cT1cN0)**	Not recommended	A workup can be performed in the case of HER2+ BC and TNBC^a^	Not recommended	Not applicable
	**IIA (cT1cN1 or cT2cN0)**	Optional	A workup can be performed whatever the BC subtype^a^	A workup can be performed in T1 N1 disease of stage IIA^c^	Not applicable
	**IIB or III**	Recommended	A workup can be performed whatever the BC subtype	A workup can be performed	•Minimal staging work-up for ABC includes a history and physical examination, hematology and biochemistry tests and imaging of the chest, abdomen and bones. •In NST ABC, [^18^F]FDG PET/CT may be used (instead of and not in addition to CT scans and a bone scan). •In invasive lobular breast cancer, CT and bone scans or whole-body MRI are preferred. •Brain imaging should not be routinely performed in asymptomatic patients.
	**IV**	Recommended	A traditional workup is recommended •Chest diagnostic CT ± contrast •Abdominal ± pelvic diagnostic CT with contrast or MRI with contrast •Brain MRI with contrast if suspicious CNS symptoms •Spine MRI with contrast if back pain or symptoms of cord compression •Bone scan or sodium fluoride PET/CT •Useful in certain circumstances^b^: [^18^F]FDG PET/CT (consider [^18^F]FES PET/CT for ER + disease) •X-rays of symptomatic bones and long and weight-bearing bones if abnormal on bone scan	•The minimum imaging work-up for staging M1 disease includes CT of the chest and abdomen + bone scan. •[^18^F]FDG PET/CT may be used instead of CT and bone scan. •Brain imaging may be considered according to BC subtype if the presence of CNS metastases alter the choice of therapy. •There is no evidence that any staging or monitoring approach provides an overall survival benefit over another.	

a In the NCCN guidelines, [^18^F]FDG PET/CT is most beneficial and accurate for advanced disease (stage III) and invasive ductal (compared to lobular) histology, but may be useful in selected circumstances of earlier stage disease (stage IIA disease: T1N1, T2N0) such as: equivocal CT + bone scan results; suspicion of undetected nodal and/or distant disease; and treatment response assessment. An [^18^F]FDG PET/CT may be utilized as an adjunct to, or in lieu of, initial standard staging and may be performed simultaneously with diagnostic CT. Conversely, a bone scan or sodium fluoride PET/CT may not be needed if an upfront [^18^F]FDG PET/CT clearly indicates consistent findings on both PET and CT components.

b In the NCCN guidelines, circumstances in which [^18^F]FDG PET/CT can be useful are not detailed.

c for cN+, large tumors (>5 cm), aggressive biology and in clinical signs, symptoms or laboratory values suggesting the presence of metastases. This means at least N1 and/or T3 disease (table-1).

d ABC (Advanced breast cancer) comprises both inoperable locally advanced breast cancer (LABC) and metastatic breast cancer (MBC).

In early BC, ESMO [9,10] recommends routine staging evaluation directed at locoregional disease, but does not support the use of [^18^F]FDG PET/CT in the staging of locoregional disease, due to its limited sensitivity when compared with the gold standard, sentinel lymph node biopsy and axillary lymph node dissection. In the EANM-SNMMI guidelines, [^18^F]FDG PET/CT is not recommended in stage I BC [3]. The ESMO guidelines [9,10] state that asymptomatic distant metastases are rare, and that most patients do not benefit from comprehensive laboratory tests and radiological staging. Staging, including CT-chest, abdominal imaging (US, CT or MRI) and bone scan can be considered for patients with: clinically positive axillary nodes; large tumors (e.g., >5 cm); aggressive biology; clinical signs/symptoms; or laboratory values suggesting the presence of metastases. This includes any ≥ N1 or ≥ T3 disease, which encompasses T0/T1 disease with clinically involved nodes (excluding those that are cN0 but staged pN1 by axillary surgery), as well as all stage IIB-III BC (Table 1, Table 2). According to ESMO, [^18^F]FDG PET/CT may be useful when conventional methods are inconclusive. It can also replace traditional imaging for staging in high-risk patients. However, in cases of ILC and low-grade tumors, [^18^F]FDG PET/CT may be less sensitive [9].

In a 2020 study [43], among 196 patients with BC, the overall upstaging rate to stage IV based on finding unsuspected distant metastases on [^18^F]FDG PET/CT was 14 % (27/196); 0 % for stage IIA, 13 % for stage IIB (10/79), 22 % for stage IIIA (9/41), 17 % for stage IIIB (5/30), and 37 % for stage IIIC (3/8). [18F]FDG PET/CT had comparable costs to conventional imaging panel and results in lower radiation dose exposure [43]. In another multicenter study published in 2020, [^18^F]FDG PET/CT reduced false-positives by half, minimized the workup for incidental findings, and allowed for earlier treatment initiation [44]. [^18^F]FDG PET/CT was cost-effective, and at one institution, cost-saving [44]. These two studies add financial and radiation protection data to support the use of [^18^F]FDG PET/CT in the baseline staging of BC patients (instead of a conventional imaging panel). A prospective, randomized clinical trial published in 2023, analyzed 369 patients with stage IIB (T3N0, but not T2N1) or III NST BC, staged with [^18^F]FDG PET/CT or conventional imaging (bone scan, CT chest/abdomen/pelvis) [45]. [^18^F]FDG PET/CT identified more distant metastases than conventional modalities, upstaging 12 % more patients (23 % vs 11 %) to stage IV. Consequently, this changed therapy decisions and reduced the number of patients initially considered for multi-modality (chemotherapy, surgery, and radiotherapy) curative intent therapy [45]. These 3 studies [[43], [44], [45]] also support the joint EANM-SNMMI guidelines [3] for workup of clinical stage IIB or higher BC with a single modality: [^18^F]FDG PET/CT.

#### 2.1.2 Staging advanced/metastatic breast cancer

In advanced clinical stage IV BC, the joint EANM-SNMMI expert panel recommends [^18^F]FDG PET/CT for determining the precise extent of metastatic disease and to improve treatment planning [3]. As recommended in lower stages, [^18^F]FDG PET/CT can be done instead of, and not in addition to, conventional imaging (bone scan, chest X-ray or CT-chest, liver ultrasound or CT-abdomen) [3]. Beyond the scope of this review, but included within the guidelines [3,[8], [9], [10], [11], [12], [13], [14]], is brain imaging. [^18^F]FDG PET/CT has a low negative predictive value for brain metastases detection. In general, brain MRI is the preferred imaging modality for evaluating clinical suspicion of brain metastases in patients with BC, and the role of screening for brain metastases is largely unknown.

For ABC (comprising both inoperable LABC and metastatic breast cancer [MBC]), ABC5 Guidelines recommend imaging of the chest, abdomen and bones [13]. During the ABC7 conference in November 2023, a specification was made that for NST BC [^18^F]FDG PET/CT, if available, is preferred instead of and not in addition to conventional imaging [42]. But for most ILC CT-scans and bone scans or whole-body MRI are preferred [42].

ESMO recommends CT chest-abdomen and bone scan for workup of stage IV disease. According to ESMO and ABC5 guidelines, [^18^F]FDG PET/CT may be used instead of conventional imaging, but according to ESMO, there is no evidence that any staging or monitoring approach provides an overall survival benefit over another [11,12]. ESMO and ABC5 recommend that the imaging modality chosen at baseline should be applied for disease monitoring to ensure comparability [11,[12], [13]]. However in a recent study, [^18^F]FDG-PET/CT appears a better predictor of progression-free and disease-specific survival than abdominal-chest contrast-enhanced computed tomography (CE-CT) when used to monitor MBC [46].

In stage IV disease, NCCN considers [^18^F]FDG PET/CT useful in certain circumstances, though without precise specification. NCCN recommends a panel of conventional imaging tools (Table 2), although [^18^F]FDG PET/CT detects distant metastases in a one-stop-shop with a sensitivity and specificity of 100 % and 96.4 %, respectively, versus a sensitivity of 61.5 % and specificity of 99.2 % for conventional imaging [47].

### 2.2 Assessment of breast cancer recurrence

#### 2.2.1 Patient follow-up after curative-intent therapy of early-stage breast cancer

According to NCCN [8] and ESMO [10], the follow-up of an asymptomatic patient with early stage BC treated with curative-intent is based on regular physical exams and annual breast imaging, such as mammography. In the absence of clinical signs or symptoms suggestive of recurrence, there is no indication for laboratory or imaging studies for metastases screening (Table 3). Joint EANM-SNMMI Guidelines agree with this principle.

**Table 3:** Summary of the recommendations regarding the assessment of breast cancer recurrence.

Clinical scenario	Joint EANM-SNMMI Guidelines [3]	NCCN Guidelines [8]	ESMO Guidelines [[9], [10], [11], [12]]	ABC ° 5, 6 and 7 consensus Guidelines [[13], [14], [42]]
**Monitoring for BC recurrence in asymptomatic patients**	• [^18^F]FDG PET/CT is not recommended.	•Follow-up is based on regular physical exam and annual mammography. •In the absence of clinical signs and symptoms suggestive of recurrent disease, there is no indication for laboratory or imaging studies for metastases screening.	•Follow-up is based on regular physical exam and annual breast imaging [10]. •In asymptomatic patients, laboratory tests or other imaging are not recommended [10].	
**Concern forsuspicion ofBC recurrenceor** i**nitial workup of a known BC recurrence**	•[^18^F]FDG PET/CT can be recommended when there are: -signs or symptoms suggestive of metastatic disease, -laboratory suspicion of recurrence -to guide site of biopsy. •[^18^F]FDG PET/CT is useful to detect the site and extent of recurrence when conventional imaging methods are equivocal. •[^18^F]FDG PET/CT can substitute for CT and/or bone scan in the detection of bone metastases. •[^18^F]FDG PET/CT can be recommended to improve RT planning.	Imaging for systemic staging: •Chest diagnostic CT ± contrast •Abdominal ± pelvic diagnostic CT with contrast or MRI with contrast •Brain MRI with contrast if suspicious CNS symptoms •Spine MRI with contrast if back pain or symptoms of cord compression •Bone scan or [^18^F]NaF PET/CT •Useful in certain circumstances^a^: [^18^F]FDG PET/CT (consider FES PET/CT for ER+ disease) •X-rays of symptomatic bones and long and weight-bearing bones abnormal on bone scan	•In the suspicion of oligometastic disease, systemic imaging staging is indicated, preferably with [^18^F]FDG PET/CT [12] •CT of the chest and abdomen and bone scan (or [^18^F]FDG PET/CT), •Brain imaging may be considered according to BC subtype if the presence of CNS metastases alter the choice of therapy.	•[^18^F]FDG PET/CT can be used instead of CT scans and bone scan •CT scans and bone scan are also an acceptable option •If recurrence is highly suspected (i.e. symptoms or elevated tumor markers) and CT scans and bone scan fail to diagnose it, a [^18^F]FDG PET-CT should be performed •[^18^F]FDG PET-CT should be performed to confirm the diagnosis of oligometastatic disease •Brain imaging should be done only in symptomatic patients •Staging of patients with LMD should include full spine imaging with MRI with gadolinium

LMD – leptomeningeal disease.

a In the NCCN guidelines, circumstances in which [^18^F]FDG PET/CT can be useful are not detailed.

#### 2.2.2 Assessment of suspected locoregional and/or distant metastatic recurrence

**Figure 2:**
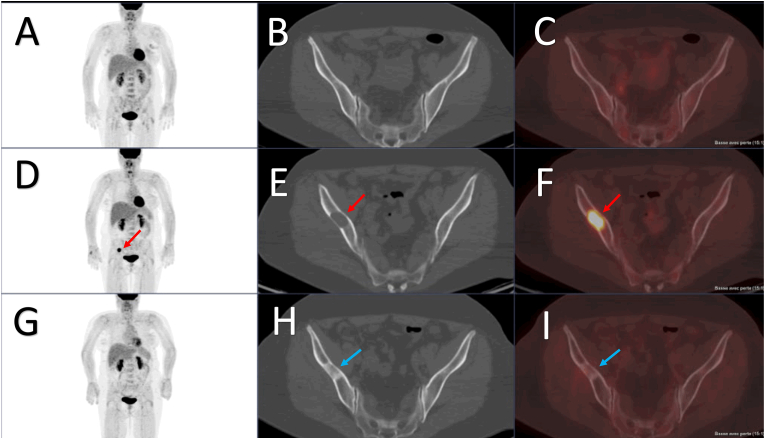
A 56-year-old woman with NST BC, grade 2, ER+, PR−, HER2- (luminal B) with confirmed axillary lymph nodes involvement was submitted to primary surgery. Baseline PET/CT staging performed after surgery showed no pathological [18F]FDG uptake (A, whole body PET MIP image, and B, C, axial CT and PET/CT fusion images of the iliac bones). Two years later, the patient was referred for a new [^18^F]FDG PET/CT examination because of isolated CEA elevation. A single lytic metastasis of the right iliac bone (red arrows) was detected (D, whole body PET MIP image; and E, F, axial CT and PET/CT fusion images of the iliac bone). The patient was treated with stereotaxic radiotherapy and targeted therapy. Six months later, PET/CT showed a complete metabolic response (G, H, I). The focus of the right iliac bone was replaced by a non-[^18^F]FDG-avid osteosclerosis (blue arrows) suggestive of a healed lesion (H and I, axial views of CT and PET/CT fusion image).

Early detection and staging of recurrence are essential for optimal management. [^18^F]FDG PET/CT imaging offers high sensitivity in detecting BC relapse [[48], [49], [50], [51], [52], [53], [54], [55], [56], [57], [58], [59], [60], [61], [62], [63]] with higher performance than conventional imaging, whether suspected by clinical examination, conventional imaging, or tumor marker elevation (CA 15.3 or CEA) (Fig. 2). Five meta-analyses showed the high performance of [^18^F]FDG PET(/CT) to detect recurrent BC [[64], [65], [66], [67], [68]]. In the meta-analysis by Pan and colleagues, MRI and [^18^F]FDG PET(/CT) were more effective than ultrasound and CT [65]. In a meta-analysis by Pennant and colleagues, [^18^F]FDG PET/CT imaging had significantly higher sensitivity than CT but the difference in specificity was not significant [66]. Neither meta-analysis showed a significant difference between [^18^F]FDG PET/CT and MRI.

[^18^F]FDG PET/CT is effective in detecting distant metastases and also in showing locoregional recurrence, especially in the chest wall and axillary and extra-axillary LN regions, and can differentiate radiation plexitis from locoregional recurrence [67,68]. Several studies have shown that [^18^F]FDG PET/CT is more effective than CT or MRI in detecting LN recurrence [52,53]. Schmidt et al. showed that [^18^F]FDG PET/CT was more sensitive than whole-body MRI for detecting LN involvement, however, whole-body MRI was somewhat more sensitive for detecting distant metastases [52]. In asymptomatic patients with increasing tumor markers and negative conventional imaging, [^18^F]FDG PET/CT has shown recurrence earlier than conventional imaging in several studies [[53], [54], [55],^59^,^66^,[69], [70], [71]]. With clinical suspicion of relapse, [^18^F]FDG PET/CT can reveal recurrence, even with negative tumor markers [72]. Compared with conventional imaging, [^18^F]FDG PET/CT offers a whole-body approach to determine the extent of disease. It improves prognostic stratification by distinguishing patients with isolated locoregional recurrence from those with distant metastases [52,54,58,59].

Joint EANM-SNMMI guidelines state that [^18^F]FDG PET/CT is useful in the detection of site and extent of recurrence when conventional imaging methods are equivocal [3]. According to the guidelines, [^18^F]FDG PET/CT can be recommended in patients: with signs or symptoms suggestive of metastatic disease; with rising serum tumour markers; to guide biopsy site; and to improve RT planning (Table 3). [^18^F]FDG PET/CT can substitute for CT and/or bone scan in the detection of bone metastases [3].

For any patient who develops clinical, laboratory, or radiographic signs or symptoms of possible metastatic disease, ESMO, NCCN and ABC consensus guidelines for the workup of recurrent MBC are similar to guidelines for stage IV baseline staging (see Table 3). If there is suspicion of oligometastatic disease, ESMO and ABC consensus guidelines recommend whole body staging, preferably with [^18^F]FDG PET/CT [12,13,42].

### 2.3 Assessment of breast cancer treatment response

#### 2.3.1 Primary systemic therapy response assessment

Primary systemic therapy (PST) is offered in many patients with stage II-III BC. This strategy allows more patients to undergo breast and axillary conserving surgery and increases the likelihood of surgery in case of inoperable primary disease; it also provides valuable information about chemotherapy efficacy. The degree of pathological response measured at surgery can determine additional adjuvant therapy. Early assessment of response to PST provides potentially useful information, as it can theoretically reduce toxicity of ineffective chemotherapy or allows for refinement of treatment. There is consensus that the gold standard imaging methods for assessing locoregional response to PST are breast-dedicated imaging modalities.

A number of studies have demonstrated the effectiveness of [^18^F]FDG PET/CT in the early assessment of PST response in mixed BC subtypes [[73], [74], [75], [76], [77], [78], [79], [80], [81], [82], [83], [84], [85], [86], [87], [88], [89], [90], [91], [92], [93]]. Taking into account the BC subtype, [^18^F]FDG PET/CT has shown good performance in predicting early pathological complete response (pCR) in TNBC [[92], [93], [94], [95], [96], [97], [98], [99], [100], [101]] and HER2+ BC [95,[102], [103], [104], [105], [106], [107]], pCR being associated with a better survival [108]. In 78 patients with TNBC, the change in the primary tumor maximum Standardized Uptake Value (SUV_max_) after two cycles of PST strongly correlated with pCR and the risk of recurrence [94]. In 2 large multicenter trials, PST for HER2+ BC was modified on the basis of early assessment by [^18^F]FDG PET/CT, with encouraging results [[105], [106], [107]].

Joint EANM-SNMMI guidelines consider [^18^F]FDG PET/CT may be used to assess early metabolic response in non-MBC, particularly in TNBC and HER2+ BC [3]. Currently optimal [^18^F]FDG PET/CT parameters to define response in the PST setting remain uncertain [[109], [110]]. In most studies, a cut-off for the reduction in the primary tumor SUV_max_ value (ΔSUV_max_) has been used to discriminate metabolic response from non-response. Unfortunately, the optimal cut-off varied between the studies, according to the BC subtype and the treatment used.

At the end of PST, several studies have shown [^18^F]FDG PET is not very sensitive in revealing residual primary tumor tissue. [^18^F]FDG PET/CT shows a tendency toward underestimation of residual tumor, and MRI performs better in this indication. Joint EANM-SNMMI guidelines do not give a specific recommendation for the use of [^18^F]FDG PET/CT in detecting residual primary tumor at the end of the PST [3]. However, EANM-SNMMI consider [^18^F]FDG PET/CT can be useful at the end of PST to perform a whole-body examination to exclude metabolically active regional LN or distant metastases before breast surgery.

ESMO does not provide specific recommendations about imaging during or at the end of PST. In the PST setting, the NCCN considers the accurate assessment of primary breast tumor or regional LN response to preoperative systemic therapy to be difficult. This assessment should include physical examination and the same imaging studies (mammogram and/or breast ultrasound and/or breast MRI) that were abnormal at the time of initial tumor staging. According to NCCN, [^18^F]FDG PET/CT is not indicated in the PST setting [8].

#### 2.3.2 Metastatic disease response assessment

Early response to treatment is also important in MBC to maximize efficacy of cancer-directed therapy. In MBC, local treatments such as surgery, radiation therapy and radiofrequency may also be used, especially in patients with oligometastatic disease. It is important to be able to utilize these treatments at the most appropriate time and to be able to evaluate their effectiveness at an early stage. Changes in metabolic activity usually occur earlier than changes in tumor size. [^18^F]FDG PET/CT has been shown to be very effective in assessing the response to therapy of patients with MBC [40,[111], [112], [113], [114], [115], [116], [117], [118], [119], [120], [121], [122], [123], [124], [125], [126], [127], [128], [129]], especially in assessing the response of bone lesions (Fig. 2) [[40], [129]]. For metabolic response criteria (particularly in MBC), EANM-SNMMI guidelines consider [^18^F]FDG PET/CT should be reported according to PERCIST or to the EORTC PET response criteria [[130], [131]]; in patients on immunotherapy, [^18^F]FDG PET/CT should be reported according to the respective EANM guidelines [[132], [133]].

According to ESMO, [^18^F]FDG PET/CT might provide earlier guidance in monitoring bone-only/predominant metastases. Prospective trials are, however, needed to study the impact on treatment decisions and overall survival (Table 4). The ABC consensus guidelines [13,14] provide specific recommendations for specific sites of metastases, and recommends: a) radiological assessment in patients with persistent and localized pain due to bone metastases to determine whether there are pathological fractures; b) neurological symptoms/signs which suggest the possibility of spinal cord compression must be investigated as a matter of urgency. This requires a full radiological assessment of potentially affected area as well as adjacent areas of the spine. MRI is the method of choice; c) MRI in patients with neurological symptoms to evaluate the possibility of brain and leptomeningeal disease. Regarding frequency of evaluation, these guidelines [13] recommend to evaluate the response to therapy for metastatic disease every 2–4 months for endocrine therapy or after 2–4 cycles for chemotherapy. Imaging of a target lesion may be sufficient in many patients and less frequent monitoring is acceptable in patients with indolent disease [13]. Nevertheless, if disease progression is suspected or new symptoms appear, additional testing should be performed in a timely manner, irrespective of planned intervals. Moreover, heterogeneity of response between metastases has been observed [129].

**Table 4:** Summary of the recommendations regarding the assessment of breast cancer treatment response.

	Joint EANM-SNMMI Guidelines [3]	NCCN Guidelines [8]	ESMO Guidelines [[9], [10], [11], [12]]	ABC ° 5, 6 and 7 consensus Guidelines [[13], [14], [42]]
**Primary systemic therapy (PST) setting**	•[^18^F]FDG PET/CT may be used to assess early metabolic response in non-metastatic BC, particularly in TNBC and HER2+. •No specific recommendation for the use of [^18^F]FDG PET to search residual primary tumour is given. •[^18^F]FDG PET/CT can be useful at the end of PST to exclude metabolically active regional lymph nodes or distant metastases before breast surgery.	The accurate assessment of in-breast tumour or regional lymph node response to preoperative systemic therapy should include physical examination and performance of breast imaging studies. MRI is more accurate than mammography for assessing tumour response to PST. The use of MRI is optional and is not universally recommended by experts in the field.	No specific recommendations about imaging are given by ESMO (during and at the end of the PST)	Not applicable
**Metastatic setting**	•[^18^F]FDG PET/CT may play a role, particularly in assessing bone metastases and enabling early response to treatment evaluation	•Frequency of monitoring: - CT Chest/Abdomen/Pelvis with contrast and bone scan: baseline prior to therapy and every 2–4 cycles for chemotherapy and every 2–6 months for endocrine therapy. -PET/CT: As clinically indicated^a^ -Brain MRI with Contrast: As clinically indicated	•The interval between imaging and treatment start should be ≤ 4 weeks. •Evaluation of response should generally occur every 2–4 months depending on disease dynamics, location, extent of metastasis and type of treatment. •If progression is suspected, additional tests should be carried out in a timely manner irrespective of planned intervals. •Repeat bone scans are a mainstay of evaluation for bone-only/predominant metastases •PET/CT might provide earlier guidance in monitoring bone-only/predominant metastases,	•Evaluation of response to therapy should generally occur every 2–4 months for endocrine therapy or after 2–4 cycles for chemotherapy, depending on the dynamics of the disease, the location and extent of metastatic involvement and type of treatment. •Imaging of a target lesion may be sufficient in many patients. In patients with indolent disease, less frequent monitoring is acceptable, but if progressive disease or new symptoms appear, additional testing should be performed in a timely manner.

a In the NCCN guidelines, circumstances in which [18F]FDG PET/CT can be useful are not detailed.

In MBC, the NCCN considers the same method of assessment should be used over time (e.g., abnormality found on chest CT should be monitored with chest CT). The NCCN recommends objective and widely accepted criteria for response, such as the Response Evaluation Criteria In Solid Tumors (RECIST) guidelines [134] and the WHO criteria [135]. According to the NCCN, functional imaging modalities, such as radionuclide bone scan and [^18^F]FDG PET/CT imaging, are particularly challenging for assessing therapy response. With bone scans, responding disease may result in a flare or increased uptake, which can be misinterpreted as disease progression, especially on the first follow-up bone scan after initiating a new therapy. For NCCN, [^18^F]FDG PET/CT is challenging due to the lack of a reproducible, validated, and widely accepted set of standards for assessing disease activity [8]. In contrast the EANM-SNMMI guidelines advocate the use of PERCIST or EORTC response criteria [3].

## 3 Discussion

Using [^18^F]FDG-PET/CT in the management of BC patients has some limitations and challenges. The technique lacks sensitivity for small tumoral tissue (primary or secondary lesions less than 5 mm are source of false negative findings) and for certain tumor characteristics such as low-grade tumors, well differentiate luminal tumor and lobular histological type [136].

PET is less sensitive and accurate than MRI for delineating the primary tumor volume and assessing multifocality [137,138]. Due to the limited spatial resolution of whole body PET systems, better performance to detect the primary tumor is expected with PET/MRI imaging [139,140], as well as with high resolution positron emission mammography (PEM) imaging [141]. Because of partial volume effect, the sensitivity of PET is low for small lymph node metastases and micrometastases [47,[142], [143], [144], [145], [146], [147], [148], [149]]. In a meta-analysis of 19 studies (1729 patients), the sensitivity and specificity of PET to detect axillary involvement were 66 % and 93 %, respectively [146]. In another meta-analysis of 62 studies (10,374 patients), the sensitivity and specificity for detecting ALN metastases were, respectively, 51 % and 100 % for US, 83 % and 85 % for MRI, and 49 % and 94 % for PET [147]. For assessing axillary status, PET does not appear to be superior to US [148] or MRI [149]. PET/MRI may in the future outperform MRI in detecting lymph node involvement [150,151]. In summary, the spatial resolution of PET imaging is insufficient for depicting small axillary lymph node metastases, especially with small primary tumors. [^18^F]FDG-PET/CT is suboptimal compared with sentinel lymph node biopsy [143]. The case is different in large, advanced or inflammatory breast tumor, especially to show lymph node involvement outside axillary level I or II [18,21,30,41].

[^18^F]FDG-PET/CT has limited performance in staging the lobular histological type. Analysis of CT findings of the PET/CT images can help detect lesions with low or no [^18^F]FDG uptake [6,7]. In a study of 146 patients with infiltrating lobular carcinoma, PET/CT revealed distant metastases (confirmed by biopsy) in 12 cases; in 3 of these 12 patients, the metastases had no FDG uptake and were seen only on the CT component of the examination [6].

To overcome these limitations, tracers other than [^18^F]FDG can be used or are currently being evaluated. These include [3]: 16α-18F-Fluoro-17β-fluoroestradiol ([^18^F]FES), [^18^F]Sodium fluoride (NaF), [^18^F]Fluciclovine (FACBC), Fibroblast activation protein inhibitor (FAPI), and Human epidermal growth factor receptor-2 (HER2) targeted agents. New instruments based on PET imaging such as PEM, PET/MRI and high-resolution digital PET/CT are also designed to improve the performance of sensitivity and spatial resolution of conventional PET/CT.

[^18^F]FDG-PET/CT has also been highlighted as an expensive technique. However in a study of 196 breast cancer patients [43], the cost of [^18^F]FDG-PET/CT in the staging of breast cancer was comparable to that of conventional workup (based on CE-CT of the chest, abdomen, and pelvis, and the addition of bone scintigraphy) [43] and in an another multicenter study of 564 patients [^18^F]FDG-PET/CT was cost-effective and, at one institution, was shown to be cost-saving [44].

Despite the well-known limitations, [^18^F]FDG-PET/CT has demonstrated its superiority over other imaging techniques and its value in optimizing treatment for stage IIB or higher NST BC. [^18^F]FDG-PET/CT is useful to detect the site and extent of suspected recurrent BC and may play a major role in monitoring treatment response. Considering PET scanners are becoming widely available and allow for whole-body and fast evaluation of patients, providing information that significantly impacts clinical management, it is expected that its use will increase in the future to better support clinical decisions.

Among international recommendations, the Joint EANM-SNMMI guidelines more widely recommend the use of [18F]FDG PET/CT in patients with BC compared to NCCN, ESMO or ABC guidelines. Although there are overlaps among EANM-SNMMI, NCCN, ESMO and ABC recommendations, there are many differences which are specific to each guideline. It should be noted that the EANM-SNMMI guideline is the most recently published guideline, featuring a systematic literature search making use of the AGREE tool [152]. According to official criteria, the level of evidence was determined, and consensus was reached regarding the level of recommendation for each statement [152].

Regarding initial staging, [^18^F]FDG PET/CT is recommended by the EANM-SNMMI as a first-line modality from stage IIB up to and including stage IV and may be useful in certain cases for stage IIA. In NCCN, ESMO, and ABC guidelines, [^18^F]FDG PET/CT is most often recommended as complementary to conventional imaging, and rarely in place of it. However, according to ESMO and ABC, [^18^F]FDG PET/CT can replace conventional imaging for staging high-risk and MBC.

When assessing BC recurrence during routine follow up, NCCN and ESMO only recommend diagnostic imaging if there is suspicion of recurrence. Similarly, EANM-SNMMI states that [^18^F]FDG PET/CT is useful to detect the site and extent of recurrence when there is either clinical or laboratory suspicion of recurrence, or when conventional imaging methods are equivocal. Furthermore, if oligometastatic disease is suspected, ESMO and ABC guidelines recommend whole body staging, preferably with [^18^F]FDG PET/CT.

Finally, when evaluating PST response, EANM-SNMMI state that [^18^F]FDG PET/CT may be used to assess early metabolic response, particularly for HER2+ BC and TNBC, and for whole body assessment at the end of PST. In contradiction, according to NCCN and ESMO, [^18^F]FDG PET/CT has no indication in the PST setting. In the metastatic setting, EANM-SNMMI state that [^18^F]FDG PET/CT may play a role in monitoring treatment response, mainly to evaluate bone metastases and to determine early response to treatment. According to ESMO, [^18^F]FDG PET/CT might provide earlier guidance in bone-only/predominant metastases. In MBC NCCN recommends using CT with RECIST 1.1 or WHO criteria to assess response to therapy, while EANM-SNMMI advocates the use of [^18^F]FDG PET/CT with PERCIST or EORTC criteria.

This review of international guidelines on the role of [^18^F]FDG PET/CT in NST BC was elaborated by a multidisciplinary team of experts in BC and is the first document providing a comprehensive and up-to-date summary about this topic. The major limitation is the fact that it was written by the same authors of the EANM-SNMMI guidelines and some also participated in the clinical oncology guidelines.

Future perspectives include the analysis of PET quantification as a possible tumor biomarker. Additionally, the definition of which specific PET radiopharmaceutical should be use depending on the breast cancer subtype remains to be clearly defined. Randomized, multi-center trials across BC subtypes have demonstrated prolonged disease-free and overall survival for advanced breast cancer since the introduction of new systemic treatments [[153], [154], [155]], possibly changing the way that oligometastatic disease (OMD) may be managed in the future [156]. As such, it is vital for partnerships between nuclear medicine and medical/surgical/radiation oncology to consider the optimal role of [^18^F]FDG PET/CT in future trial designs in patients with advanced disease, and OMD in particular. Furthermore, the current data comparing the diagnostic performance of [^18^F]FDG PET/CT and [^18^F]FDG PET/MRI showed promising results with PET/MRI demonstrating higher sensitivity (0.87 vs 0.81) and area under the curve value (0.98 vs 0.95), with similar specificity (0.97 vs 0.97) and lower radiation dose exposure to the patient (∼50 %) [[157], [158], [159]]. Depending on availability and costs, this technology may also improve some limitations and impact the clinical management of patients with BC.

## 4 Conclusion

There are some agreement and many differences between EANM-SNMMI, NCCN, ESMO and ABC recommendations, which are specific to each guideline. Not surprisingly, the joint EANM-SNMMI guidelines more widely recommend the use of [^18^F]FDG PET/CT in patients with BC. The evident consensus between these guidelines is related to the need for imaging studies when there is clinical suspicion of BC recurrence. The main similarities between EANM-SNMMI, ESMO and ABC are found in initial staging due to the notion that [^18^F]FDG PET/CT can replace conventional imaging for staging high-risk or metastatic patients with breast, and in the early assesment of bone metastases where [^18^F]FDG PET/CT may be useful. Increased use of PET to support clinical decisions about patients with BC is foreseen, therefore, well-designed trials with multidisciplinary collaboration are needed to clearly define the position of PET in the management of patients with breast cancer.

The recently published EANM-SNMMI guidelines [3] are already endorsed by several oncology and imaging societies: ACR, ESSO, ESTRO, EUSOBI/ESR, and EUSOMA. This, as well as multi-disciplinary evidence generation, may foster optimal use of [^18^F]FDG PET/CT for patients with breast cancer and lead to greater harmonization of imaging and clinical guidelines in the future.

## Funding

This research did not receive any specific grant from funding agencies in the public, commercial, or not-for-profit sectors.

## Declaration of competing interest, conflicts of interest

Gary Cook: research grant from Breast Cancer Now and he was a previous member of their scientific advisory board.

Gary Ulaner: Consultant/Advisory Board/Research Funding – GE Healthcare, Lantheus, ImaginAb, Point Biopharma

Heather Jacene: Blue Earth Diagnostics, honoraria and research support; Consulting, advanced accelerator applications, spectrum dynamics,

royalties: Cambridge University Press; all are not related to the work presented in this manuscript.

Philip Poortmans: medical advisor of Sordina IORT Technologies S.p.A., not related to the work presented in this manuscript.

Ritse Mann: research grants from/with Beckton and Dickinson, Siemens, Bayer Healthcare, Screenpoint medical, Koning, and PA Imaging, and is a medical advisor to Screenpoint, Bayer, Guerbet, and BD. All are unrelated to the work in this manuscript.

Fatima Cardoso: Personal financial interest in form of consultancy role for: Amgen, Astellas/Medivation, AstraZeneca, Celgene, Daiichi-Sankyo, Eisai, GE Oncology, Genentech, Gilead, GlaxoSmithKline, Iqvia, Macrogenics, Medscape, Merck-Sharp, Merus BV, Mylan, Mundipharma, Novartis, Pfizer, Pierre-Fabre, prIME Oncology, Roche, Sanofi, Samsung Bioepis, Seagen, Teva, Touchime. Institutional financial support for clinical trials from: Amgen, Astra-Zeneca, Boehringer-Ingelheim, Bristol-Myers-Squibb, Daiichi-Sankyo, Eisai, Fresenius GmbH, Genentech, Gilead, GlaxoSmithKline, Ipsen, Incyte, Nektar Therapeutics, Nerviano, Novartis, Macrogenics, Medigene, MedImmune, Merck, Millenium, Pfizer, Pierre-Fabre, Roche, Sanofi-Aventis, Sonus, Tesaro, Tigris, Wilex, Wyeth.

Given her role as Editor-in-Chief, Fatima Cardoso had no involvement in the peer-review of this article and had no access to information regarding its peer review.

All the other authors declare no conflict of interest.

## Ethical approval

Approval was not required.

## CRediT authorship contribution statement

**David Groheux:** Writing – review & editing, Writing – original draft, Visualization, Validation, Supervision, Resources, Project administration, Methodology, Investigation, Formal analysis, Data curation, Conceptualization. **Sofia C. Vaz:** Writing – review & editing, Writing – original draft, Visualization, Validation, Supervision, Project administration, Methodology, Formal analysis, Data curation, Conceptualization. **Philip Poortmans:** Writing – review & editing, Writing – original draft, Visualization, Validation, Project administration, Methodology, Conceptualization. **Ritse M. Mann:** Writing – review & editing, Writing – original draft, Validation. **Gary A. Ulaner:** Writing – review & editing, Writing – original draft, Validation. **Gary J.R. Cook:** Writing – review & editing, Writing – original draft, Validation. **Elif Hindié:** Writing – review & editing, Writing – original draft, Validation. **John Patrick Pilkington Woll:** Writing – review & editing, Validation. **Heather Jacene:** Writing – review & editing, Validation. **Isabel T. Rubio:** Writing – review & editing, Validation, Writing – review & editing, Validation, Conceptualization. **Marie-Jeanne Vrancken Peeters:** Writing – review & editing, Validation, Writing – review & editing, Validation, Conceptualization. **Elizabeth H. Dibble:** Writing – review & editing, Writing – original draft, Visualization, Validation, Supervision, Methodology, Investigation, Formal analysis, Conceptualization. **Lioe-Fee de Geus-Oei:** Writing – review & editing, Writing – original draft, Visualization, Validation, Supervision, Software, Project administration, Methodology, Investigation, Conceptualization. **Stephanie L. Graff:** Writing – review & editing, Writing – original draft, Visualization, Validation, Supervision, Project administration, Methodology, Investigation, Formal analysis. **Fatima Cardoso:** Writing – review & editing, Writing – original draft, Visualization, Validation, Supervision, Resources, Project administration, Methodology, Investigation, Formal analysis, Data curation, Conceptualization.
